# Next-generation nephrology: part 2—mainstreaming genomics in nephrology, a global perspective

**DOI:** 10.1007/s00467-025-06711-7

**Published:** 2025-02-28

**Authors:** Asheeta Gupta, Kushani Jayasinghe, Amar Majmundar, Nina Mann, Rajiv Sinha, Matthew G. Sampson, Catherine Quinlan

**Affiliations:** 1 Dept. of Pediatric Nephrology, Birmingham Children’s Hospital, Birmingham Women’s and Children’s NHS Foundation Trust, Birmingham , UK; 2 Dept of Pediatric Nephrology, , Melbourne, Australia, Royal Children’s Hospital, Melbourne, Australia; 3 Kidney Regeneration, Murdoch Research Institute, Melbourne, Australia; 4 University of Bristol, Bristol, UK; 5 Dept of Nephrology, Monash Medical Centre, Melbourne, Australia; 6 Monash University, Melbourne, Australia; 7 Melbourne Health, Melbourne, Australia; 8 Division of Pediatric Nephrology, Boston Children’s Hospital, Massachusetts, USA; 9 Harvard Medical School, Massachusetts, USA; 10 Institute of Child Health, Kolkata, India; 11 Brigham and Women’s Hospital Kidney Disease Initiative, Broad Institute, Massechusetts, USA; 12 Dept of Pediatrics, School of Medicine, University of Melbourne, Melbourne, Australia; 13 Dept of Pediatric Nephrology, Great Ormond Street Hospital for Children NHS Foundation Trust, London, UK

**Keywords:** Kidney, Genetics, Genomics, Clinic, Mainstreaming, Education, Equity

## Abstract

Kidney genetic services are being created worldwide, revolutionising the way in which we manage families with suspected monogenic kidney disease. There is potential to learn from one another, whether one is just embarking on this journey or within an established kidney genetics service model with aspirations to optimise it further. This concluding portion of our two-part educational review explores the global efforts to integrate genomics into nephrology. We discuss key considerations for establishing kidney genetics services and share insights from successful implementation in Australia, India, the United Kingdom (UK) and the United States (US), through case studies. Widespread integration of genomics within nephrology still faces barriers including limited genomics education among clinicians, high costs and ethical concerns. Educational strategies including workshop-based, online resources and clinical decision tools are aiming to address the genomic literacy gap among nephrologists. Multidisciplinary kidney genetics clinic models comprising nephrologists, geneticists, clinical scientists and counsellors are proving to be an effective model of delivering this diagnostic tool. Data of how kidney genetics clinics can foster collaboration with registries to facilitate research and shared learning to optimise care for patients are becoming evident. We also explore the importance of equitable access to genomics services across diverse populations, advocating for policies that address disparities in access to healthcare and genetic data representation. We hope to highlight the importance of upskilling the nephrology workforce to fully leverage the advances in genomic medicine and ensure comprehensive, accessible and personalised care for patients with genetic kidney diseases.

**Graphical abstract:**

**Figure:**
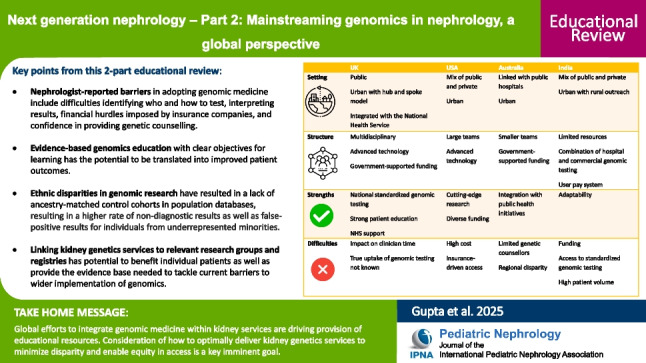
A higher resolution version of the Graphical abstract is available as Supplementary information

**Supplementary Information:**

The online version contains supplementary material available at 10.1007/s00467-025-06711-7.

## Introduction

An evidence base for the diagnostic and clinical usefulness or utility of genomic sequencing for the diagnosis of suspected genetic kidney diseases is building. Part 1 of this two-part review expanded on this, justifying why and how genomic testing should be used as a diagnostic tool for patients with suspected genetic kidney disease. Molecular diagnoses can provide further clinical insight and inform therapeutic planning and counselling for patients and their families [1–4]. Understanding the range of genomic test modalities, as well as selecting the most appropriate test for the patient, interpretation and communication of results following genomic sequencing are some of the key skills nephrologists need to know. An understanding of the cost-effectiveness of this diagnostic tool was also reviewed in part 1, which is relevant for all healthcare systems globally.

Bringing genomics to the forefront of clinical care is a political priority in many jurisdictions, with plans to mainstream this technology over the next decade [5–7]. For those that have achieved this already, it has meant creating bespoke models tailored to the needs of their jurisdiction. But the rate of implementation has been slower than expected. Barriers to integration of this technology differ within countries and across the world. A common hurdle for nephrologists is that the understanding of genomics, poised to redefine the field, has been largely insufficient. Most nephrology curricula require a comprehensive understanding of kidney pathology, dialysis and transplantation. In contrast, only a few have learning objectives and education that address incorporation of genomic medicine in clinical practice. For the second part of our mainstreaming genomics in nephrology educational review, we outline steps towards the creation of a kidney genetics service, key considerations, educational resources and existing models including international case studies highlighting the experience of the authors in establishing dedicated kidney genetics clinics in the US, UK, India and Australia.

### Implementation of genomic medicine and the service design process

Integration of genomic medicine into healthcare or the process of mainstreaming aims to enable non-geneticists in primary and secondary care to incorporate this field into their clinical practice. This includes acquiring specific skills, such as taking a family history, accurate phenotyping and who to test. Key additional skills include determining a testing strategy, ordering tests, interpreting results and counselling of patients and families pre- and post-test [8]. Mainstreaming offers a unique opportunity to complement kidney services, especially those with established subspecialised domains, such as transplant or glomerular nephrology. By integrating genomic testing into routine workflows, early identification of genetic diagnoses can facilitate timely referral to the appropriate specialist team, inform management decisions for patients and their families and aid access to treatments. This synergy underscores the potential of mainstreaming genomics, not only to broaden diagnostic capabilities but also to support highly specialised pathways to deliver high-quality kidney care.

The process of mainstreaming has lagged the pace at which genomic research has evolved. Additionally, implementation of genomics has differed drastically between and within countries [9, 10], prompting deep dives into potential causes. A recent rapid review analysed barriers to implementing genomic programmes from 30 international studies [11]. Unsurprisingly, the most prevalent obstacle in two-thirds of cases was a lack of genomic education and literacy, with a need for significant upskilling of the healthcare workforce. Similar themes were found from a systematic review analysing mainstreaming in the hospital setting [12]. Nurses and physicians displayed low confidence in performing genetics-related tasks. This included obtaining a family history, performing risk assessments, appropriate referrals for genomic testing/review, interpreting test results and poor engagement in discussions related to genomic testing, suggesting an awareness of their limited knowledge. The significant costs of enacting system-wide changes were reported as a barrier in just under half of all studies [11]. This encompassed the running costs of testing, data analysis, additional time and staff needed to provide education, training, consent and counselling. Additional costs of incorporating and maintaining genomic information and technology within healthcare systems added to this burden. Lack of integration between electronic health records and genomic data was thought to cause further dispersal in already fragmented healthcare systems. Reluctance to reimburse costs of testing due to the lack of available evidence which met healthcare payer thresholds for clinical validity and utility was also raised [13, 14].

Ethical, legal and psychosocial issues surrounding the handling and implications of genetic information can be challenging topics to manage and influence healthcare practitioners’ own motivation for utilising this technology within their practice [12]. There is a perception that genetic information may inflict psychological harm to patients, despite genetic counselling demonstrating an ability to reduce anxiety and improve accuracy of genetic risk [15]. The time and counselling skills required to support parents’ emotional and psychosocial needs in stressful intensive care settings have been reported as a barrier to implementing rapid genomic sequencing [16]. Avoiding discussions to explore patients’ and families’ concerns around genomic testing may mean vital opportunities to obtain family history or a DNA sample that could benefit relatives may be missed.

Sharing of genomic data is necessary to improve our understanding of the genetic basis of disease and in turn patient care. However, concerns relating to insurance or employment discrimination resulting from inappropriate sharing of genetic information remain [12]. Ethical and legal safeguards for individuals and families undergoing genomic testing are key. Lack of guidelines, regulations and standards are significant barriers that require government and policy-making support for genomics [11, 12]. Furthermore, the lack of clarity on how national and international general data protection regulation laws apply to data sharing in relation to genetic or genomic data is proving to be another barrier to the effective sharing of information [17].

### Attitudes of nephrologists towards implementing genomic medicine

Valuable work exploring the attitudes of nephrologists towards implementing genomic medicine has reinforced the themes listed above and added a speciality-specific dimension [18]. A survey of Australian adult, paediatric and trainee nephrologists revealed that 37% had never requested a genomic test. Ranking the most challenging aspects of undertaking genomic testing revealed the most difficult was the selection of the correct test, followed by interpreting results, identification of suitable patients to investigate, genetic counselling for the family, giving results, integrating results into clinical care, ordering tests and ending with the least challenging, consenting for the genetic test. Follow-on work targeting the views of 25 nephrologists revealed negative attitudes towards genomic testing. This was due to a perceived lack of clinical impact of results, funding for tests and clinical processes. Other reasons for not pursuing testing included a lack of genomic literacy, long wait times and difficulties interpreting genetic results [19]. Many expressed they were unprepared to use genetic and genomic data and felt there were time barriers in integrating genomics. Concerns that the volume and nature of big data may grow beyond the interpretive capacity of physicians and malpractice liability were also raised.

Barriers to genomic implementation were frequently interrelated and varied according to geographical location, but as many have concluded, tackling one would not be sufficient to guarantee implementation [12]. A holistic approach has been suggested to address micro- and macro-level implementation challenges.

Frameworks and outcome measures to aid change would need to be applied from the initial research stages through to the development and integration of genomic medicine. Once genomic medicine programmes are integrated into healthcare settings, continued evaluation and adaptation to ensure sustainable implementation have also been recommended [20]. Facilitators for the successful implementation of genomics include access to policy frameworks, regulations, guidelines, clinical decision support tools, genetic counselling support with education and training for healthcare staff [11].

## Optimising genomic education and impact on clinical implementation

Provision of adequate education and support to improve awareness and knowledge is a fundamental part of implementing change in healthcare organisations and, as such, is not a new concept. A potentially novel challenge is ensuring educational efforts keep up with the breadth, complexity and pace at which genomic knowledge is changing, whilst also covering key underlying concepts for those completely naive to the topic. It is recognised that genomics education needs to be incorporated into medical school curricula and post-graduate training programmes. However, the navigation of a complex landscape of regulatory systems that govern education in each locality is adding delay. At present genomic literacy is not addressed as part of the US board examination for paediatric nephrology [21] but is part of the speciality training curriculum for adult kidney medicine published by the Joint Royal College of Physicians Training Board for Europe and the UK [22]. In addition, generic core competencies for post-graduate, non-genomic physicians in the UK have been formulated according to the level of involvement or specialisation in genomic medicine [23].

There are numerous genomic education programmes being created for the post-graduate setting in a variety of formats and healthcare settings. It is known that quality evidence-based education with clear objectives for learning has the potential to be translated into improved patient outcomes [24]. The RISE2 reporting standards [24, 25] support the development of an evidence base for genomics education by facilitating transparency and appraisal of interventions. As there is no overarching governing body to publish or mediate the use of reporting standards for genomic education interventions, global and national organisations are needed to help increase awareness and encourage their use [24]. Similar efforts in incorporating education around HIV and AIDS into medical, nursing, primary and secondary school curricula were driven by bodies such as the World Health Organization [26, 27] as well as changes in policy and legislation. In the UK, the introduction of the 1993 Education Act granted schools more control over their curriculum and autonomy in addressing public health concerns, such as HIV and AIDs. Similar support for the introduction of education around the concept of patient safety in medical school curricula has been driven by national and international bodies [28, 29].

Healthcare professionals have expressed a preference for genomic education that demonstrates clinical utility via workshops, lectures, conferences or online materials [12]. Recognition that genomic medicine training will be an ongoing, career-long learning process that needs to be tailored to individual needs has influenced the development of a wealth of educational resources. The UK-based Genomic Education Programme offers stand-alone small-scale sessions, self-directed certified short courses as well as provision of clinical and teaching resources [23]. Formal higher education in genomic medicine can also be sought through several academic institutions through this programme. Furthermore, the 3-year post-graduate education programme provided by ERKnet has been developed with a kidney genetic focus [30].

Educational opportunities incorporated into the clinical setting can offer consistent, collaborative, multidisciplinary learning. This approach encourages learning alongside effective clinical practice whilst maintaining a flexible and constructive use of healthcare professionals’ time using in-person interactions, case-based learning or interactive discussion. Strategies applied to genomics education include ‘flipped classroom’ blended learning [31], hands-on or experiential education [32] and embedding trained genomic specialists within mainstream clinical settings to provide on-hand education [33]. International projects (100KGP, INGNITE) utilised webinars, conference calls and presentations between the teams involved in the programmes to foster engagement, collaboration and feedback from clinicians and hospital management, to concurrently improve and modify outcomes [34, 35]. An Australian study measuring collective ties between clinicians showed that “hands-on learning” and “making group decisions” were the most potent influences on their genomic practice. This was achieved by strategically building a genomic learning community by creating boundary-spanning roles [34]. Thus, education programmes are inherently linked to implementation strategies, and teamworking in this manner also facilitates improvement in other parallel areas such as coordinated care pathways and ethical issues surrounding genomic medicine.

Concurrent, active involvement of diverse groups of patients in implementation strategies through education programmes, patient advisory boards or through the co-design of information materials has also been shown to enhance the success of this process [33]. Patient advisory boards are structured groups of patients, caregivers or representatives who provide insights, feedback and advice to the kidney genetics team and hospital managers. Participants can be individuals with lived experience of the kidney genetics service or those who are completely naïve, representatives from patient organisations or caregivers/relatives of patients. The focus of their involvement may be to evaluate a service model and help to identify unmet needs or gaps in care. This approach aims to utilise a transparent and inclusive decision-making process that is centred around the patient and their family.

Delivery of medical education has transformed with advances in IT and increased accessibility through wireless technologies and smartphones. Online genomic learning tools that improve accessibility internationally and facilitate sharing of educational efforts have been advocated [31]. Free open-access medical (FOAM) education resources arose with the goal of making education accessible to all. Don’t Forget the Bubbles (DFTB) [36] is a popular FOAM resource within paediatrics and includes sessions introducing key concepts in genomics.

Nephmadness has addressed a number of genomics-related topics over the last 10 years [37]. An array of podcasts covering different aspects of genomics are also emerging: ‘Genepod’ from Genetics in Medicine [38], ‘Genomics Now’ and ‘The G Guide’ by Genomics England [39]. However, it is important to note that there is no formal peer review process, and therefore, critical appraisal by the user is required. Incorporating genomics training within established conference settings is already underway and has been successfully delivered as part of the ASN 2023 (Genetics in Clinical Nephrology Programme), and in the upcoming IPNA pre-course on inherited kidney diseases [40].

### The kidney genetics clinic model

**Fig. 1:**
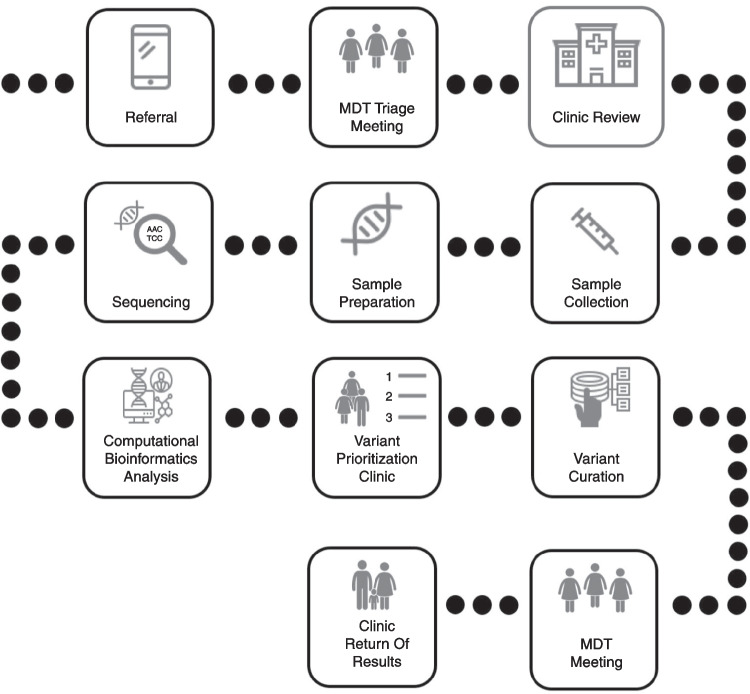
Example of a kidney genetics clinic workflow

Kidney genetics clinic structures appear to have variations of the hub and spoke model where expert centres play a role in supporting colleagues to consider and pursue genetic or genomic evaluations [41, 42]. A national kidney genetics clinic model based in Israel aims to be a one-stop shop to aid diagnostics with priority access to those with high suspicion of monogenic kidney disease based on family history, extra-renal involvement, unexplained or known subtypes of CKD (chronic kidney disease) and those with severe or atypical presentations [43]. Key aspects of this model include joint phenotyping between nephrology and genetics specialities with the creation of a bespoke phenotype–driven list of genes prioritised for analysis prior to testing. The process of phenotyping encompasses collating an individual’s set of observable characteristics within the kidney, such as cystic kidneys, and/or outside the kidney such as retinitis pigmentosa. Adult and paediatric collaborations seen in this and other models provide family-centred care within a single clinic [44]. Family-centred care is a patient-focused approach which aims to acknowledge the vital role families play in the health and well-being of patients, especially children with chronic disease. These care models incorporate ways to promote effective communication and collaboration with members of the family whilst maintaining respect for their cultural beliefs and preferences in care decisions. Genetic results may have a direct and indirect impact on the health of family members other than the proband. Family-centred kidney genetics clinics aim to address these issues to facilitate active family involvement, satisfaction and improved healthcare outcomes [45]. Alternative models utilise the skillset of individuals within the multidisciplinary team; allocating return of result, consent to undertake research and cascade testing (genetic testing in at-risk relatives to look for a disease-causing variant already known to affect a family member) to genetic counsellors versus a collection of medical and family history, risk assessment, testing strategy and informed consent to a multidisciplinary team composed of an adult nephrologist and genetic counsellor [46]. Some models have successfully used telemedicine to run consultations, with in-person appointments made available on request [46]. Other kidney genetics clinics formalise their input and utilise it at key points such as at the point of triage, variant prioritisation and again at the point of receiving results prior to their disclosure [1] (Fig. 1).

### Role of decision support tools in genomic medicine

Clinical decision support tools can help clinicians with limited genomic knowledge by providing ‘just-in-time’ information with links to evidence [47, 48]. These tools can guide assessments or provide recommendations at the point of care based on clinical management guidelines, best practice and/or research evidence. Tools such as GeNotes, a UK-based, ‘just-in-time’ web resource, are designed for health care professionals to access in the clinic setting to help guide them through the processes of selecting an appropriate genomic test to understanding the result and communicating it to the patient [48]. The resource spans adult and paediatric indications as well as subspecialities such as nephrology.

Key outcomes from a mixed-methods systematic review of clinical decision support tools in genomic practice included improved recognition and assessment of patients at increased risk of inherited kidney disease with increased uptake of genomic testing [49]. The majority of clinical decisions were made in line with appropriate guidelines with high rates of clinician satisfaction [49]. Clinical decision support tools cannot and are not intended to replace assessment and management by those with genomic or genetic expertise. Rather, these tools have utility in supporting non-geneticists to appropriately access or incorporate genomic testing in their practice.

### Composition of a kidney genetics multidisciplinary team

Given the need for detailed phenotyping and interpretation of genomic test results, patients may benefit from an assessment by a dedicated kidney genetics service with the involvement of a multidisciplinary team: a clinical geneticist, genetic counsellor and laboratory scientist along with a paediatric or adult nephrologist. The multidisciplinary model is particularly useful in obtaining a detailed family history, accurate phenotyping, and discussing the intricacies of genomic testing that come with obtaining consent and delivering results [50, 51]. Few nephrologists have trained in the explanation of the advantages and disadvantages of testing and may not be aware of the practical considerations of ordering the test, including limitations and costs of the various genomic tests available, which is a core skillset for genetic counsellors and clinical geneticists. Similarly, they are unlikely to be confident in explaining future reproductive options [52] or the implications of secondary findings [53]. On the other hand, few clinical geneticists or genetic counsellors will be able to discuss the management implications of autosomal recessive polycystic kidney disease or the potential consequences of being a female with a disease-causing variant in *COL4A5*-associated Alport syndrome. With much of clinical nephrology delivered by a multidisciplinary team of nurse specialists, dieticians, pharmacists, psychologists and social workers, nephrologists are culturally well placed to sit as part of a multidisciplinary kidney genetics team together with a genetic counsellor and clinical geneticist.

**Fig. 2:**
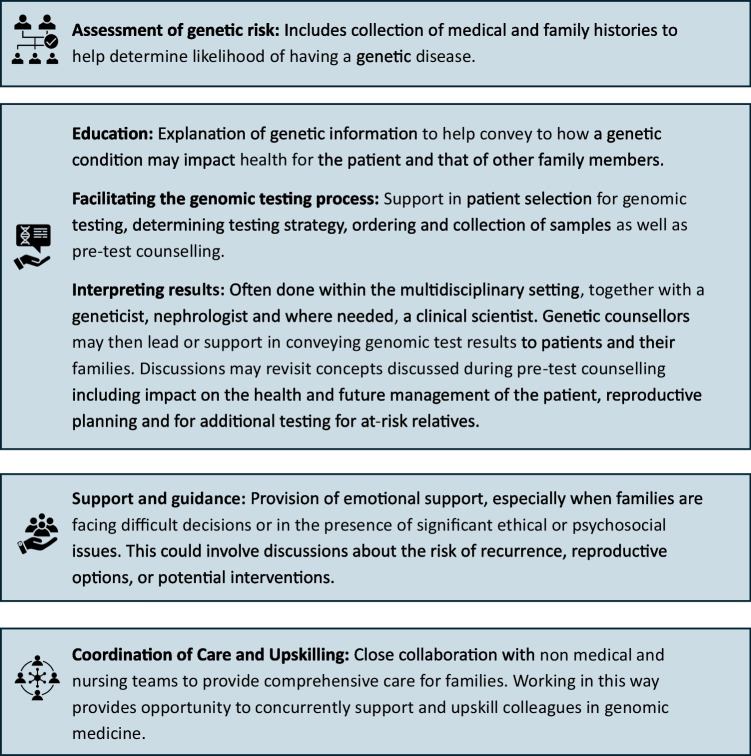
Summary of skills genetic counsellors can offer to assist with the genomic testing process [54]

Genetic counsellors are a group of accredited and regulated professionals with specific training and expertise in genomic medicine and counselling skills [54, 55]. Tasks genetics counsellors may undertake can be wide-ranging, often reflective of the needs of the service as well as their experience and expertise (Fig. 2). In some jurisdictions, they can act as on-call specialists for urgent referrals and triage referrals into services [55]. They are the main health professional group who ‘takes care of the family’, facilitating family communication, coping and adjustment as well as cascading information and arranging testing of at-risk relatives. A key role that distinguishes genetic counsellors is that of being patient advocates both within the clinical genetics setting but more so in their work across specialities where the best interest of the patient and/or the family may find itself in conflict with what is deemed ‘routine practice’. In 2019, there were an estimated 7000 genetic counsellors practicing across 28 countries [56].

### Receiving genomic test results outside of clinical practice

Genomic testing may be undertaken in settings other than clinical practice. Though this is uncommon, nephrologists may be asked to advise on such results and should either possess the skillset to comment on their clinical validity or know where to refer patients for further discussion and clinical testing.

Direct-to-consumer genomic sequencing, such as that offered by companies such as 23andMe and AncestryDNA, is marketed as providing insights into ethnicity and family background. However, some provide third-party analysis of raw data to derive health information [57]. Although not marketed as a clinical test it may be perceived as such by patients anxious for a diagnosis, who present to their treating clinician with results that appear to be diagnostic. Unfortunately, with 40% of results shown to be false positive [58], they should be viewed with scepticism and should be confirmed by clinical testing with appropriate genetic counselling. In resource-limited environments, research level testing may be offered to patients despite not being clinically accredited. Though not optimal, the use of research testing can be pragmatic, particularly when the clinician has awareness of its limitations and can inform the patient that the test results are not clinically accredited. Since variant analysis at a research level may fall below the standards required for a clinical diagnosis, these results should be viewed as an indicator rather than a definitive diagnosis and patients should be counselled accordingly. Positive results should be validated in a clinical laboratory and negative results should not be assumed to be set in stone.

### Contributing to research within clinical models

The integration of genomics into routine nephrology practice has been supported by the work of many clinician scientists, and these individuals continue to generate new data and drive the development of this new sub-speciality. Thus, research is integrated into many genetic kidney services. Functional data is crucial for cases where in silico (predictive information gathered using computer modelling) information cannot provide enough evidence to determine whether a variant is causative. Many functional studies are undertaken in the research setting, highlighting the benefit of creating links to access groups with disease-specific expertise [59, 60].

Worldwide kidney registries aim to systematically collect, analyse and report information about kidney disease in a standardised way such as RaDaR (National Registry of Rare Renal Disease) [61], ERKReg (European Registry for Rare Kidney diseases) [62] and the ANZDATA registry [63]. The scope of registry data has broadened over time, from describing the characteristics and epidemiology of kidney disease to spatial distribution and temporal trends in treatment and outcomes to support the assessment of treatment effectiveness. Registry data is driving service improvements by monitoring quality of care, benchmarking, standardising therapies/approaches informing public health strategies, enabling pay-for-performance models for financing care and conducting economic evaluation studies.

Gaps in understanding aspects of monogenic CKD, such as its true prevalence across different populations and evidence of causality may be achieved through expanding the remit of registries to include genetic data which can then be utilised in the research setting, whilst protecting the privacy of research projects. This will facilitate the assembly of larger global cohorts with genetically defined kidney disease for both research and clinical trials. Producing data which analyse the impact of genetic or genomic testing on short- and longer-term clinical outcomes for patients with CKD, cost-effectiveness and the impact of centres of expertise on the provision of high-quality care for these patients are just some of the key priorities proposed by KDIGO [64].

### Funding issues related to genomic testing

One of the major hurdles to implementing widespread genomic medicine is cost. In the US, with a predominantly privatised health insurance system, funding issues abound due to difficulty in getting medical insurance approval. This involves numerous administrative steps for the clinical provider. For example, letters of medical necessity or discussions with insurance-associated physicians can help improve the chance of insurance approval. However, if genetic or genomic testing does not meet a pre-specified list of requirements, these steps may not improve the outcome. This is further complicated by the patchwork nature of the medical insurance marketplace in the US, where insurance companies have non-overlapping standards for what testing is approved. Increasing data demonstrating the cost-effectiveness of genomic sequencing [65] should over time improve the access to such testing modalities as this evidence is presented to insurers during the approval process.

Even with insurance approval, the co-pay fee or remaining uncovered cost of testing in the US can be prohibitive for some families. Industry-sponsored testing does exist. This together with research-based genomic testing are considered in cases where there is no insurance approval or when costs remain high.

### Case studies and global models of service delivery

**Fig. 3:**
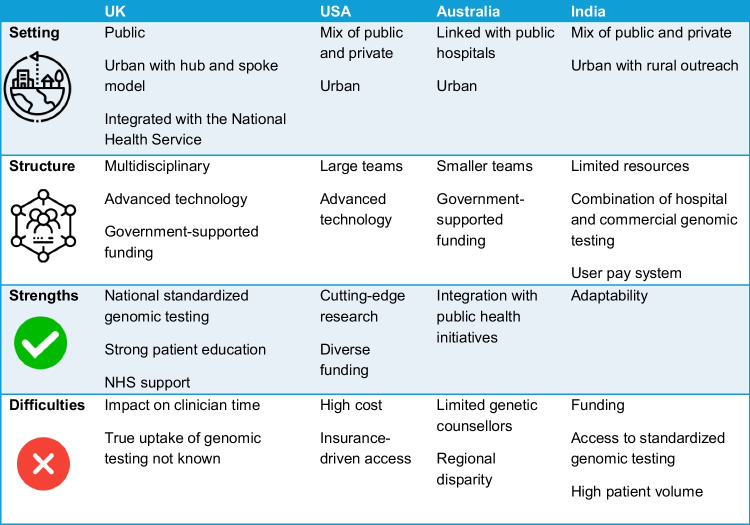
Organizational structure of kidney genetics services for different global settings based on the author’s experiences

Here the authors reflect on their experiences of creating kidney genetics services within their jurisdiction. Comparison of services highlights the differences in healthcare settings which impacts how services are structured and the difficulties they face (Fig. 3).

### USA

A kidney genetics clinic consisting of two nephrologists and a genetics counsellor with expertise in inherited kidney disease and variant interpretation was recently established at Boston Children’s Hospital in Boston, Massachusetts. Patients are referred by their primary nephrologist or general paediatrician for genetic counselling and initiation of testing when deemed appropriate.

Pre- and post-test genetic counselling is provided to review the risks and benefits of genetic or genomic testing, potential for unexpected results or variants of uncertain significance (variants where there is uncertainty whether they are disease-causing) necessitating further investigation, implications for insurance and test results. Indication-driven testing encompassing primarily panel-driven tests is performed based on the individual’s clinical presentation and the pre-test probability of identifying a specific genetic disorder.

Multidisciplinary team meetings are held monthly to review test results, assign variant pathogenicity (the process of determining whether the variant is disease-causing) and decide on appropriate management steps. This includes referral to additional subspecialty services in cases where extra-renal manifestations are common, or when cascade testing is needed. When cascade testing does reveal a pathogenic variant in adult family members, collaborations with local adult kidney genetic programmes has helped to facilitate referrals to ensure that these individuals receive appropriate nephrology care.

As mentioned above, insurance coverage for genomic testing in the United States has substantially improved in recent years but remains a major barrier to the implementation of this testing for many families. Prior authorisation is necessary for most private insurances and can lead to a delay in genomic testing as insurance approval is sought. Public payers, on the other hand, are more likely to approve insurance coverage for genomic testing, but the out-of-pocket costs for some families can still be prohibitive. Our hospital has a created a dedicated patient financial services team with expertise in the financial landscape of genomic testing, which has greatly facilitated the prior authorisation process and has alleviated some of the administrative burden for providers. Future studies that demonstrate the clinical benefit and cost-effectiveness of genomic testing amongst patients with kidney disease will hopefully help to overcome existing barriers in this process.

### India

Kidney genetics clinics within the Institute for Child Health, a government-funded teaching hospital in Kolkata, were created in May 2022. This clinic structure utilises a multidisciplinary model including a genetic counsellor skilled in bioinformatics as well as the kidney team. The clinic is run through a government hospital, but genetic or genomic tests are paid for by the patient and family. Referrals are made from around the region of West Bengal. Genetic or genomic testing is comporised of direct-to-consumer testing as well as tests requested through the clinic that are undertaken in commercial laboratories. The main issues faced within the clinic model are the costs associated with travelling to the clinics and genetic or genomic testing. Genomic testing strategies have therefore moved to undertake a single, broader test (whole exome sequencing with reanalysis where needed) to minimise clinic visits and overall cost. Many families do not opt for cascade testing for at-risk family members, mainly due to cost. Collaboration and support from the established kidney genetics service at Birmingham Children’s Hospital, UK, was facilitated through the International Society of Nephrology Sister Centre program. Guidance and education were provided through monthly case-based discussions with attendance of the kidney genetics multidisciplinary team from both sites including trainees and those who wish to upskill. Educational opportunities were developed through the same collaboration, which led to the creation of a national Indian kidney genetics workshop linked to the Indian Society of Paediatric Nephrology meeting in 2023, followed by a regional workshop for Kolkata in 2024.

### UK

**Fig. 4:**
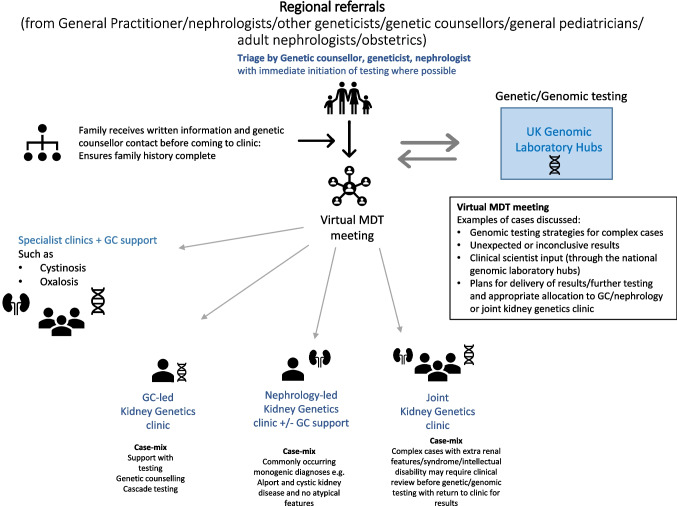
Kidney genetics clinic model at Birmingham Children’s Hospital UK

The paediatric kidney genetics service at Birmingham Children’s Hospital was established in January 2018 to incorporate genomic testing. The service trialled different models of service delivery with funding from Genomics England to serve as a vanguard for similar clinics in the UK. The proposed hub and spoke model of delivery meant the tertiary nephrology centre (hub) provides clinical support and guidance to the region including smaller peripheral centres (spokes) through dedicated kidney genetics clinics and associated multidisciplinary meetings. The core team consists of a paediatric nephrologist, geneticist and genetic nurse specialist with later funding for a genetic counsellor. The main hurdle faced by the service was the sheer volume of referrals which affected wait times. Altering the clinic model helped to overcome these issues. Changes were based on identification of the most common reason for referral and design of streamlined patient pathways for those indications such as persistent microscopic haematuria, cystic kidney disease and cascade testing. These pathways offered the referrer guidance or genomic counsellor (GC) support to reduce waiting times for testing or obtaining results. Furthermore, creating additional clinics that were led by a GC, the kidney team or a combination for a pre-defined cohort deciding at the point of triage was possible as skills and confidence across both teams grew. Finally, the kidney genetics model included multidisciplinary meetings to triage new referrals, discuss testing strategies and variant interpretation and decide on approaches for communicating results to families. Over time, these multidisciplinary team meetings had more emphasis and were attended by trainees as an educational tool (Fig. 4). The creation of a national clinical group, RenGenUK, has offered much-needed dissemination of new knowledge, collaboration and case-based learning for established kidney genetic multidisciplinary teams in the UK. Attendees include nephrologists, geneticists, genetic counsellors and clinical scientists. This is an incredibly useful resource which is driven and delivered by clinical need but like similar initiatives remains unfunded.

### Australia

A dedicated paediatric kidney genetics service was established at the Royal Children’s Hospital in Melbourne, Australia, in 2016 as a multi-disciplinary service where children with suspected genetic disease were reviewed by a paediatric nephrologist, clinical geneticist and genetic counsellor. Over 500 children have had whole exome sequencing with targeted analysis of a phenotype-driven gene list through the service since its establishment. The service was initially funded with support from philanthropy and a research grant from Melbourne Genomics that expanded the clinic model into state-wide adult nephrology services in Victoria, Australia. Published outcomes from the kidney genetics service in Victoria demonstrated a high diagnostic yield, significant clinical utility and substantial health economic impact of sequencing for patients with kidney disease [1, 66] and supported an application for federal funding of genomic sequencing for patients with suspected monogenic kidney disease.

In removing one barrier to access, federal funding for genomic sequencing introduced another as referrals have increased beyond the capacity of the dedicated kidney genetics clinic. In response to this challenge, the combined adult and paediatric kidney genetics service in Victoria is moving to a mainstreamed model of care. Paediatric and adult nephrologists will be supported to provide genetic sequencing to less complex patients through the general nephrology clinics, with embedded genetic counsellors, a dedicated education programme and the use of novel decision aids and communication tools. More complex cases will continue to be reviewed in the kidney genetics multidisciplinary clinic as the team will take on a broader consultative role within the general nephrology team. Outcomes from this study will be available later in 2025.

### Equity, diversity and inclusion

As genomic medicine becomes more integrated within nephrology care, it is essential for us to make concerted efforts to ensure equity in the access to and interpretation of genomic testing across different socioeconomic and ethnic groups. Prior studies have shown that not only are underrepresented minorities less likely to have access to genetics, but also that there is a higher rate of inconclusive results when genomic testing is performed [67–69].

Many of the barriers that contribute to the disparities in access to genetics clinics are consistent with those that exist across healthcare disciplines, including referral bias, complexities within the scheduling process itself and the ability to attend clinic appointments [70]. Indeed, it has been shown that those who attend a genetics clinic visit have higher levels of education and income and are less likely to identify as being from minority racial groups [71]. Given the documented benefits of genomic testing in establishing a diagnosis and potentially leading to directed therapies, addressing these inequities will be imperative to reduce the already existing disparities in health outcomes. Countries with socialised healthcare such as the UK and Australia offer funded access to genomic testing for patients with kidney disease which should reduce these disparities.

Unique to genomic testing is the importance of having ancestry-matched control cohorts in the interpretation of test results. Due to the historic ethnic disparities that have existed in research studies, it has been found that there is both a higher rate of non-diagnostic results as well as false positive results for individuals from underrepresented minority groups [69, 72]. As one example, common variants amongst African Americans in hypertrophic cardiomyopathy genes were initially misclassified as pathogenic due to the paucity of diverse control data [72]. Efforts such as the Genome Aggregation Database (gnomAD), which includes over 70,000 genomes across diverse ancestries have sought to overcome some of these limitations. However, the ongoing inclusion of diverse ethnic groups in research efforts will be critical to ensure that there is a broad and equal benefit of genomic testing.

## Conclusion

To take advantage of the advances in genomics, a competent and skilled workforce of clinicians formally trained in genomics is required. But despite intensive care physicians being at the forefront of this revolution, even they report lower confidence with genomic sequencing compared to genetics professionals, with 77% of intensivists favouring a clinical genetics-led service delivery model for genomic sequencing [73]. This lack of confidence is widespread, with most specialists reporting that they do not feel competent in identifying inheritance patterns, interpreting genomic test results or incorporating these results into their patients’ care. Even among those who have recently completed nephrology training, half do not feel competent to utilise genomic medicine in their practice [74].

Though there is an obvious need for educational interventions, this is happening in an uncoordinated fashion reaching the enthusiastic minority rather than the majority. As genomics becomes as much a part of nephrology as histopathology, dialysis and transplant, enthusiasm is not enough. Only a minority of educational interventions in genomics are led by instructors with educational qualifications [25], and there is a lack of reporting standards to assess the educational interventions that are available. We suggest that professional societies should offer practical training in kidney genetics at educational meetings for their general membership and that training programmes should ensure that newly qualified nephrologists are competent to assess which patients could benefit from kidney genetics assessment. Pre-courses are running at ESPN, IPNA and ASN.

Given the complexity and the lack of formal training in clinical genomics for virtually all nephrologists practicing today, we advocate for the use of a multidisciplinary kidney genetics clinic model to maximise the impact and utility of genomic testing for our patients. This model is likely always going to prove superior, but our hope would be that there is an upskilling of the existing workforce of nephrologists to play a larger role in leading this delivery of complex care required in patients with suspected genetic disease. Clinical nephrologists are used to working in large multidisciplinary teams, whether within a dialysis unit or a transplant clinic. Thus, in preparing to implement clinical genomics in nephrology, it should not prove disruptive to add genetic counsellors, clinical geneticists and genetic nurse specialists [75] to teams already consisting of doctors, specialist nurses, dieticians, psychologists, pharmacists, social workers and families. Audits of the clinical genetic healthcare workforce have shown that our colleagues are motivated and prepared to embrace new models of care [76]: now paediatric nephrologists need to rise to this challenge.

## Key summary points

1. The aspects of genomic testing that remain barriers for nephrologists include identifying appropriate patients for genomic testing, knowing which genomic test to order, interpreting genomic results, surmounting financial hurdles imposed by insurance companies, and providing genetic counselling.
2. Evidence-based genomics education with clear objectives for learning has the potential to be translated into improved patient outcomes.
3. Ethnic disparities in genomic research have resulted in a lack of ancestry matched control cohorts in population databases, resulting in a higher rate of non-diagnostic results as well as false-positive results for individuals from underrepresented minorities.
4. Linking kidney genetics services to relevant research groups and registries has the potential to facilitate benefits for individual patients as well as provide the evidence base needed to make changes to some of the current barriers to the wider implementation of genomics.

## Multiple choice questions

Answers appear following the References.

1. Which of these statements is true
  - Underrepresented minorities are more likely to have false positive genomic testing results.
  - Underrepresented minorities are just as likely as individuals from well-represented ethnic groups to attend a kidney genetics clinic.
  - Underrepresented minorities are less likely to have a positive genetic result.
  - There is equal ethnic representation in large population databases like gnomAD.
2. Strategies to disseminate genomic education include:
  - Provision of webinars attended by healthcare professionals and managers
  - Team-based ‘hands-on learning’ and ‘making group decisions’
  - FOAM resources
  - Case-based learning through kidney genetic multidisciplinary meetings
  - All of the above
3. Successful implementation of genomics includes access to which of the following?
  - Access to policy frameworks, regulations, and guidelines
  - Clinical decision support tools
  - Genetic counselling support
  - Education and training for healthcare staff
  - All of the above

## Supplementary Information

Below is the link to the electronic supplementary material.

Graphical Abstract (PPTX 187 KB)

## Data Availability

All data supporting the findings of this study are available within the paper and its supplementary information.
