# Supplementary material for: Next-generation nephrology: part 2—mainstreaming genomics in nephrology, a global perspective
Source: Pediatr Nephrol. 2025 Feb 28;40(9):2779–93. doi: 10.1007/s00467-025-06711-7 (PMC12296791; doi:10.1007/s00467-025-06711-7)
Supplement: Supplementary file 1 — Graphical Abstract (PPTX 187 KB) [file 467_2025_6711_MOESM1_ESM.pptx]

## Slide 1
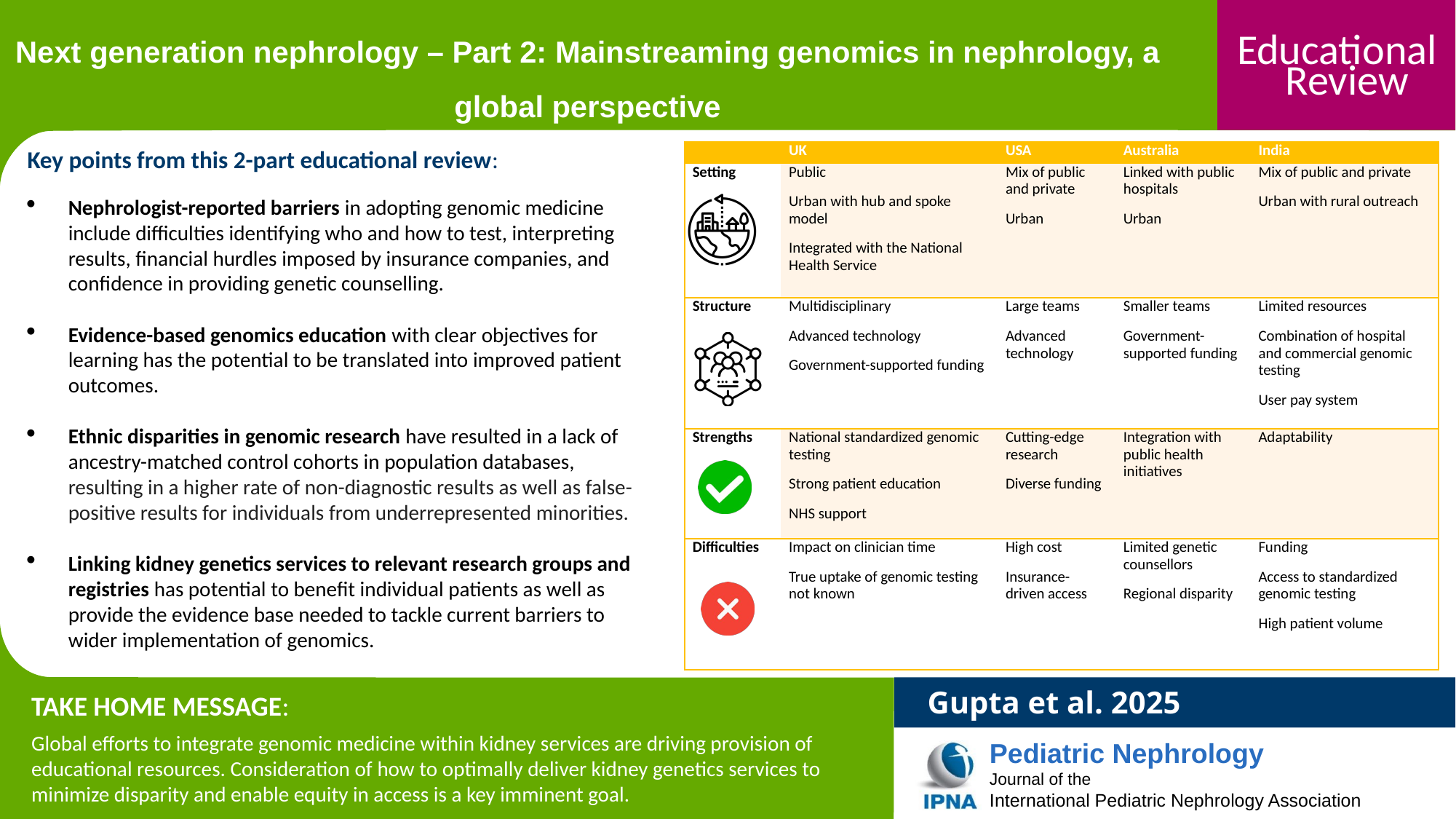

Next generation nephrology – Part 2: Mainstreaming genomics in nephrology, a global perspective
Key points from this 2-part educational review:
Nephrologist-reported barriers in adopting genomic medicine include difficulties identifying who and how to test, interpreting results, financial hurdles imposed by insurance companies, and confidence in providing genetic counselling.
Evidence-based genomics education with clear objectives for learning has the potential to be translated into improved patient outcomes.
Ethnic disparities in genomic research have resulted in a lack of ancestry-matched control cohorts in population databases, resulting in a higher rate of non-diagnostic results as well as false-positive results for individuals from underrepresented minorities.
Linking kidney genetics services to relevant research groups and registries has potential to benefit individual patients as well as provide the evidence base needed to tackle current barriers to wider implementation of genomics.
| | UK | USA | Australia | India |
| --- | --- | --- | --- | --- |
| Setting | Public Urban with hub and spoke model Integrated with the National Health Service | Mix of public and private Urban | Linked with public hospitals Urban | Mix of public and private Urban with rural outreach |
| Structure | Multidisciplinary Advanced technology Government-supported funding | Large teams Advanced technology | Smaller teams Government-supported funding | Limited resources Combination of hospital and commercial genomic testing User pay system |
| Strengths | National standardized genomic testing Strong patient education NHS support | Cutting-edge research Diverse funding | Integration with public health initiatives | Adaptability |
| Difficulties | Impact on clinician time True uptake of genomic testing not known | High cost Insurance-driven access | Limited genetic counsellors Regional disparity | Funding Access to standardized genomic testing High patient volume |
Gupta et al. 2025
TAKE HOME MESSAGE:
Global efforts to integrate genomic medicine within kidney services are driving provision of educational resources. Consideration of how to optimally deliver kidney genetics services to minimize disparity and enable equity in access is a key imminent goal.
